# Supplementary material for: End Sequence Analysis Toolkit (ESAT) expands the extractable information from single-cell RNA-seq data
Source: Genome Res. 2016 Oct;26(10):1397–410. doi: 10.1101/gr.207902.116 (PMC5052061; doi:10.1101/gr.207902.116)
Supplement: Supplemental Material [file supp_gr.207902.116_Supp_Materials.tar.gz › ESAT-esat_v0.1/doc/html/classbroad_1_1pda_1_1geneexpression_1_1_expression_experiment_info.html]

ESAT: broad.pda.geneexpression.ExpressionExperimentInfo Class Reference


ESAT


- Main Page
- Related Pages
- Packages
- Classes
- Files

- Class List
- Class Index
- Class Hierarchy
- Class Members

All Classes Namespaces Files Functions Variables Enumerator Pages

- broad
- pda
- geneexpression
- ExpressionExperimentInfo

Public Member Functions |
List of all members

broad.pda.geneexpression.ExpressionExperimentInfo Class Reference

|  |  |
| --- | --- |
| Public Member Functions | |
|  | ExpressionExperimentInfo (String line) |
|  | |
| String | getBatch () |
|  | |
| String | getExperimentBarcode () |
|  | |
| String | getPlateName () |
|  | |
| String | getExperimentPlatePosition () |
|  | |
| String | getExperimentName () |
|  | |
| String | getExperimentHybDate () |
|  | |
| boolean | passedQC () |
|  | |
| String | getSampleType () |
|  | |
| int | getTimePoint () |
|  | |
| boolean | isControl () |
|  | |

## Constructor & Destructor Documentation

|  |  |  |  |  |  |
| --- | --- | --- | --- | --- | --- |
| broad.pda.geneexpression.ExpressionExperimentInfo.ExpressionExperimentInfo | ( | String | *line* | ) |  |

## Member Function Documentation

|  |  |  |  |  |
| --- | --- | --- | --- | --- |
| String broad.pda.geneexpression.ExpressionExperimentInfo.getBatch | ( |  | ) |  |

|  |  |  |  |  |
| --- | --- | --- | --- | --- |
| String broad.pda.geneexpression.ExpressionExperimentInfo.getExperimentBarcode | ( |  | ) |  |

|  |  |  |  |  |
| --- | --- | --- | --- | --- |
| String broad.pda.geneexpression.ExpressionExperimentInfo.getExperimentHybDate | ( |  | ) |  |

|  |  |  |  |  |
| --- | --- | --- | --- | --- |
| String broad.pda.geneexpression.ExpressionExperimentInfo.getExperimentName | ( |  | ) |  |

Here is the caller graph for this function:

|  |  |  |  |  |
| --- | --- | --- | --- | --- |
| String broad.pda.geneexpression.ExpressionExperimentInfo.getExperimentPlatePosition | ( |  | ) |  |

|  |  |  |  |  |
| --- | --- | --- | --- | --- |
| String broad.pda.geneexpression.ExpressionExperimentInfo.getPlateName | ( |  | ) |  |

|  |  |  |  |  |
| --- | --- | --- | --- | --- |
| String broad.pda.geneexpression.ExpressionExperimentInfo.getSampleType | ( |  | ) |  |

|  |  |  |  |  |
| --- | --- | --- | --- | --- |
| int broad.pda.geneexpression.ExpressionExperimentInfo.getTimePoint | ( |  | ) |  |

|  |  |  |  |  |
| --- | --- | --- | --- | --- |
| boolean broad.pda.geneexpression.ExpressionExperimentInfo.isControl | ( |  | ) |  |

Here is the caller graph for this function:

|  |  |  |  |  |
| --- | --- | --- | --- | --- |
| boolean broad.pda.geneexpression.ExpressionExperimentInfo.passedQC | ( |  | ) |  |

---

The documentation for this class was generated from the following file:

- java/broad/pda/geneexpression/ExpressionExperimentInfo.java


---

Generated on Fri Aug 15 2014 15:11:00 for ESAT by  

 1.8.7
